# Particle Swarm Optimized Hybrid Kernel-Based Multiclass Support Vector Machine for Microarray Cancer Data Analysis

**DOI:** 10.1155/2019/4085725

**Published:** 2019-12-14

**Authors:** Davies Segera, Mwangi Mbuthia, Abraham Nyete

**Affiliations:** Department of Electrical and Information Engineering, University of Nairobi, Nairobi 30197, Kenya

## Abstract

Determining an optimal decision model is an important but difficult combinatorial task in imbalanced microarray-based cancer classification. Though the multiclass support vector machine (MCSVM) has already made an important contribution in this field, its performance solely depends on three aspects: the penalty factor C, the type of kernel, and its parameters. To improve the performance of this classifier in microarray-based cancer analysis, this paper proposes PSO-PCA-LGP-MCSVM model that is based on particle swarm optimization (PSO), principal component analysis (PCA), and multiclass support vector machine (MCSVM). The MCSVM is based on a hybrid kernel, i.e., linear-Gaussian-polynomial (LGP) that combines the advantages of three standard kernels (linear, Gaussian, and polynomial) in a novel manner, where the linear kernel is linearly combined with the Gaussian kernel embedding the polynomial kernel. Further, this paper proves and makes sure that the LGP kernel confirms the features of a valid kernel. In order to reveal the effectiveness of our model, several experiments were conducted and the obtained results compared between our model and other three single kernel-based models, namely, PSO-PCA-L-MCSVM (utilizing a linear kernel), PSO-PCA-G-MCSVM (utilizing a Gaussian kernel), and PSO-PCA-P-MCSVM (utilizing a polynomial kernel). In comparison, two dual and two multiclass imbalanced standard microarray datasets were used. Experimental results in terms of three extended assessment metrics (*F*-score, *G*-mean, and Accuracy) reveal the superior global feature extraction, prediction, and learning abilities of this model against three single kernel-based models.

## 1. Introduction

Cancer is a disorder caused by excessive and uncontrolled cell division in a body. A total of 9.6 million people died of cancer in 2018 [1]. As a matter of fact, death due to cancer can be reduced to nearly half if the cancer types are detected early and the right treatment administered in time. However, it is still a challenge for researchers to effectively diagnose cancer on the basis of morphological structure since different cancer types exhibit thin differences [2].

This challenge encourages application of data mining techniques, especially the use of gene expression data in determining the types of cancer cells. The level of gene expression can duly indicate the activity of a gene in a body cell based on the number of messenger ribonucleic acids (mRNAs). It is well known to contain information about the disease that may be in the gene sample, which may help experts in treating or preventing the disease [3].

Though next-generation sequencing (NGS) especially RNA-sequencing (RNA-Seq) is slowly replacing microarrays when analyzing and identifying complex mechanism in gene expression, e.g., in the gene expression-based cancer classification problem, it is relatively expensive compared to microarrays. Since microarrays have been used for a long time, there exist robust statistical and operational methods for their processing [4–13]. In addition, many significant microarray experiments have been conducted and are publicly available to the research community [14–20]. For microarrays, there exist large and well-maintained repositories that have collected these types of data for long. While the preprocessing and analysis steps of microarray data are mostly standardized, the establishment of RNA-Seq data analysis techniques is still ongoing in the field of transcriptomics. Because of these reasons, to date microarrays are still utilized in many gene expression-based cancer classification studies as presented in the most recent survey of hybrid feature selection methods in microarray gene expression for data for cancer classification [20–23].

The DNA microarray technology has the capability of determining the level of thousands of genes concurrently in a given experiment, which so far has facilitated the development of cancer classification by the use of gene expression data [4–13].

Clinical decision support is the most recent application of DNA microarrays in the medical domain. This support can take the form of disease diagnosis or predicting clinical outcomes in response to a treatment. Currently, the two major areas in medicine that are drawing much attention in this regard are management of cancer and other contagious diseases [24].

With the rapid development of artificial intelligence (AI), machine-learning algorithms such as artificial neural network (ANN), support vector machine (SVM), and k-nearest neighbor (KNN), many researchers have immensely applied them in the gene expression-based cancer diagnosis. For instance, the artificial neural networks (ANNs) have been proposed for the microarray gene classification due to their superior ability to map input-output structured data. Khan and Meltzer utilized the ANN in analyzing microarray gene data from patients with small round blue-cell tumours [9]. Bevilacqua and Tommasi developed an accurate classifier model based on the feed-forward ANN for estrogen receptor (ER) ± metastasis recurrence of breast cancer tumours [25]. Chen et al. [26] also modeled a classifier for microarray gene data using ANN ensembles that were based on filtering of samples. In all these studies, attractive classification accuracies were obtained.

Furey proposed an SVM based on a simple kernel to carry out gene expression data analysis, which turned out to perform remarkably [27]. Vanitha et al. utilized SVM alongside mutual information gained (MI-SVM) for feature selection [11]. In his research, he used various SVM models: linear SVM, radial basis function (RBF) SVM, quadratic SVM, and polynomial SVM. He further compared the results obtained from the proposed scheme with the k-nearest neighbor (K-NN) and ANN classifier results. Based on the obtained result, utilization of the MI-SVM obtained better results compared to K-NN and ANN, and even in some datasets, 100% accuracy was achieved.

Based on these previous research studies, it is evident that SVM has already made an important contribution in the field of microarray-based cancer classification. However, many researchers have pointed out that though the SVM is a promising classifier in microarray-based cancer classification, its performance solely depends on three aspects: the penalty parameter C of this classifier, the type of kernel utilized, and its parameters [28–32].

To improve the classification accuracy of the SVM classifier, some techniques have been presented to search for the optimal model parameters, such as the grid-search and the gradient descent [1]. Although these approaches have proven their effectiveness in the corresponding experiments, in most cases they fall into the local optimum point easily and have a defect of low efficiency [1, 18].

Recently, some meta-heuristic techniques, such as particle swarm optimization (PSO), genetic algorithm (GA), bat algorithm (BA), and dragonfly algorithm (DA) have attained promising results when utilized in tuning SVM classifier's parameters [18]. However, most of these research studies have not been applied to gene expression-based cancer analysis. In addition, they only focus on SVM with a single kernel function. Though some research studies [28] point out that combining multiple kernel functions can achieve better performance compared to a single kernel function, little research has provided an in-depth formulation and analysis of the performance of a multiclass support vector machine (MCSVM) with a combined kernel function. Thus, there would be a definite need to systematically study the complex optimization problem in the MCSVM classifier with a combined kernel applicable to gene expression-based cancer classification.

Considering PSO has a number of desirable properties, including simplicity of implementation, scalability of dimension, and a good empirical performance, and is computationally efficient compared to other optimization techniques [33], and there exist few studies on MCSVM classifier with combined kernels in microarray-based cancer classification, this paper proposes a novel gene expression-based cancer classification model, i.e., PSO-PCA-LGP-MCSVM. This model is based on particle swarm optimization (PSO), principal component analysis (PCA), and multiclass support vector machine (MCSVM) with a novel hybrid kernel function, i.e., linear-Gaussian-polynomial (LGP) kernel.

The objective of this research is to construct a MCSVM classifier with three different standard kernel functions (linear, Gaussian, and polynomial). Use PCA to reduce the dimensional complexity of the considered microarray datasets and optimize all the parameters of this model using PSO.

The overall structure of this paper takes the form of five chapters, including this introductory chapter. The remaining part of this paper proceeds as follows: a detailed presentation of the proposed model is presented in Section 2.  Section 3 deals with the considered cancer microarray datasets and the evaluation metrics used.  Section 4 focusses on the experimental results and discussions. Finally, conclusions and recommendations are given in Section 5.

## 2. PSO-PCA-LGP-MCSVM Principles

### 2.1. Normalization

Microarray gene expressions can differ by an order of magnitude. Thus, it is necessary to normalize these data to improve the performance of subsequent microarray data analysis stages like gene selection/feature extraction, clustering, and classification [1].

In this paper, the microarray gene expressions are linearly transformed from the interval [*X*_min_, *X*_max_]⟶[0,1] uniformly utilizing the following equation [1]:

(1) $$\begin{array}{c} X^{\prime }=\frac{X-X_{\min}}{X_{\max}-X_{\min}}, \end{array}$$

where *X*′ is the new normalized value of the gene expression level and *X* is the value of the gene expression level before normalization, while *X*_max_ and *X*_min_, respectively, declare the largest and least values of all the data in an attribute (gene) to be normalized.

Since the min-max normalization has the advantage of preserving exactly all the relationships among the original gene data values and does not introduce any bias [1], it is considered in this paper.

### 2.2. Principal Component Analysis (PCA)

One of the major challenges encountered in working with DNA microarray data is their high dimensionality that is coupled with a relatively small sample size. While there is a plethora of crucial information that can be derived from these large datasets, their high-dimensional nature can often hide the critical information. Thus, a process that can reduce the dimensionality complexity of this type of data is required. In addition, a dimensionality reduction step will minimize errors obtained in the subsequent classification stage [1, 12, 33–35].

In this paper, principal component analysis (PCA) that includes the calculation of variance of proportion for eigenvector is used. The steps of this algorithm are as follows:

- Let *X*′ (the normalized microarray gene expression data) be the input matrix for PCA. Each row vector of *X*′ represents the normalized expression gene values for each of the genes.
- Compute the mean (centroid) $\bar{X}$ of each gene *j* using the following equation where the sum goes through all *M* samples (tissues):

(2) $$\begin{array}{c} \bar{X}=\frac{1}{M}\overset{M}{\underset{i=1}{\sum }}X_{ij}^{\prime }, \end{array}$$

  where *M* is the number of tissues and *X*_*ij*_′ is gene *j* data.
(c) Compute the covariances (degree to which the genes are linearly correlated) as per the following equation:

(3) $$\begin{array}{c} C_{kj}=\frac{1}{M-1}\overset{M}{\underset{i=1}{\sum }}\left(X_{ki}^{\prime }-{\bar{X}}_{k}\right)\left(X_{ji}^{\prime }-{\bar{X}}_{j}\right), \end{array}$$

  where *C*_*kj*_ is the covariance of gene *k* and gene *j*, *M* is the number of samples (tissues),  *X*_*ki*_′ is the expression level of gene *k* in sample *i*, *X*_*ji*_′ is the expression level of gene *j* in sample *i*, $\;{\bar{X}}_{k}$ is the mean of expression levels of gene *k*, and ${\bar{X}}_{j}$ is the mean of expression levels of gene *j*.
(d) Form a covariance matrix *C* using the computed covariances and transform it into a diagonal matrix as depicted in the following equation:

(4) $$\begin{array}{c} C=\left[\begin{array}{ccc} C_{11} & C_{12} & C_{1M}\\ \vdots & \vdots & \vdots \\ C_{M1} & C_{M2} & C_{MM} \end{array}\right]\;⟶\left[\begin{array}{ccc} \partial_{1} & 0 & 0\\ \vdots & \ddots & \vdots \\ 0 & 0 & \partial_{M} \end{array}\right]. \end{array}$$

  The diagonal elements of the transformed matrix are the eigenvalues  ∂_1_,  ∂_2_,…, ∂_*M*_ which denote the amount of variability captured along a particular new dimension.
(e) Calculate corresponding eigenvectors as *ρ*_1_, *ρ*_2_,…, *ρ*_*M*_ using the following equation:

(5) $$\begin{array}{c} \partial_{k}\rho_{k}=C\partial_{k}. \end{array}$$

(f) Sort the eigenvalues in descending order, i.e., ∂_1_ ≥ ∂_2_ ≥ ∂_2_,…, ∂_*M*−1_ ≥ ∂_*M*_.
(g) The eigenvectors corresponding to the *k* largest eigenvalues (where *k* < *M*) are the first *kprincipal components.*
(h) Select the first *keigenvectors* via the cumulative proportion of variance (eigenvalues). The proportion of variance (PPV) for each principal component is determined as follows:

(6) $$\begin{array}{c} \text{PPV}=\frac{\partial_{i}}{\sum_{i=1}^{M}\partial_{i}}\times 100\%. \end{array}$$

- Form the principal component matrix *P*, a matrix consisting of selected *k* eigenvectors that correspond to the largest *k* eigenvalues, where the *k* eigenvectors are derived from eigenvalues that meet the criterion in the following equation:

(7) $$\begin{array}{c} \frac{\sum_{i=1}^{k}\partial_{i}}{\sum_{i=1}^{M}\partial_{i}}\times 100\%\;\geq \;95\%. \end{array}$$

(j) Compute dimensionally reduced microarray gene expression data *X*_DimRed_′ using the following equation:

(8) $$\begin{array}{c} X_{\text{DimRed}}^{\prime }=\;X^{\prime }\times P. \end{array}$$

Hence, the analysis reduces the highly dimensioned original microarray datasets to *P* for each sample, which are the inputs for the multiclass support vector machine (MCSVM).

To be able to measure the generalization error for each considered model, per-fold PCA was adopted. This is achieved by first conducting a separate PCA on each calibration set and then applying this transformation on the validation set. This same transformation is achieved by first subtracting the means of the calibration set from the validation set and then projecting these data onto the principal components of the training set achieved this. The underlying assumption is that the testing and training set should be derived from the same distribution, which justifies this process.

### 2.3. Multiclass Support Vector Machine (MCSVM)

The MCSVM classifier is based on Vapnik–Chervonenkis (VC) dimension of the statistical learning theory and the structural risk minimization [1, 5, 7, 11, 36].

The main objective of MCSVM is to map the preprocessed, nonlinear inseparable microarray gene expression data into a linear highly dimensioned manifold *θ* by the use of a transformation ∅:*R*^*N*^⟶*θ*, then obtaining the optimal hyperplane Ψ :  *ψ*(*x*)=(*ω* · *ϕ*(*x*)+*b*) by solving the following optimization convex problem (the soft margin problem) [36]:

(9) $$\begin{array}{c} \begin{array}{cc} \min & \left(\omega ,\xi \right)=\frac{1}{2}\omega^{2}+\beta \overset{n}{\underset{i=1}{\sum }}\xi_{i}\\ \text{Subject to} & \;y_{i}\left(\omega \cdot \phi \left(x\right)+b\right)\geq 1-\xi_{i},\;\text{for all }1\leq i\leq n, \end{array} \end{array}$$

where *ω* is a coefficient vector of the hyperplane in the manifold (feature space), *b* is the threshold value of the hyperplane,  *ξ*_*i*_ is a slack factor introduced for classification errors, and *β* is a penalty factor for errors.

The parameter *β* controls the penalty of misclassification and its value is normally determined via cross-validation. Larger values of *β* normally lead to a small margin which minimizes classification errors while smaller values of *β* may produce a wider margin resulting in many misclassifications.

The feature space *θ* is highly dimensioned, so its direct computation can lead to “dimension disaster.” However, since *ω*=∑_*i*=1_^*n*^*δ*_*i*_*y*_*i*_∅(*x*_*i*_), then all the operations of the support vector machine (MCSVM) in the feature space *θ* are only dot products. And since kernel functions, i.e., *G*(*x*_*i*_, *x*_*i*′_)=∅(*x*_*i*_) · ∅(*x*_*i*′_), are efficient at handling dot products, they were introduced into the SVM. This implies there is no need to know how to map the microarray gene expression data from its original space to the feature space *θ*. Thus, selection of a kernel and its coefficients is vital in the computational efficiency and accuracy of an MCSVM classifier model [28–32].

The common kernel functions that are utilized as continuous predictors include [1, 5, 28]:

1. Linear kernel:

(10) $$\begin{array}{c} G\left(x_{i},x_{i^{\prime }}\right)=x_{i}\cdot x_{i^{\prime }}. \end{array}$$

(2) Polynomial kernel:

(11) $$\begin{array}{c} G\left(x_{i},x_{i^{\prime }}\right)={\left(\eta *\left(x_{i}\cdot x_{i^{\prime }}\right)+\delta \right)}^{d}, \end{array}$$

  where *η* > 0, *δ* ∈ *R*, and *d* ∈ *Z*^+^.
(3) Gaussian kernel:

(12) $$\begin{array}{c} G\left(x_{i},x_{i^{\prime }}\right)=\exp\left(\frac{x_{i}-x_{i^{\prime }}^{2}}{2\sigma^{2}}\right), \end{array}$$

where *σ* > 0.

These MCSVM kernel functions can be broadly categorized as follows: local kernel functions and global kernel functions. Samples far apart have a great impact on the global kernel values while samples close to each other greatly influence the local kernel values. The linear and polynomial kernels are good examples of global kernels while the Gaussian radial basis function and the Gaussian are local kernels [28, 30–32, 37].

Relatively speaking, the linear kernel function has a better extraction of global features from samples, the polynomial kernel has good generalization ability, and the Gaussian kernel (the most widely used kernel) has a good learning ability among all the single kernel functions. Thus, it is evident that utilizing a single kernel function-based MCSVM classifier in a given application such as gene expression data may neither attain good learning ability, proper global feature extraction ability, and a better generalization capability. In trying to overcome this hiccup, two or more kernel functions can be combined [28–32].

### 2.4. Linear-Gaussian-Polynomial MCSVM (LGP-MCSVM)

In trying to build a kernel model that has better global feature extraction, good learning, and prediction abilities, the work presented in this paper combines the merits of two global kernels (linear and polynomial) and one local kernel (Gaussian). This paper therefore proposes a novel kernel “linear-Gaussian-polynomial (LGP)” kernel, which is formulated as follows:

(13) $$\begin{array}{c} G_{\text{LGP}}\left(x_{i},x_{i^{\prime }}\right)=\beta_{1}\cdot \left(x_{i}\cdot x_{i^{\prime }}\right)+\beta_{2}\cdot \;\exp\left(-\beta_{3}\cdot \left(\frac{{\left(\eta \times \left(x_{i}\cdot x_{i^{\prime }}\right)+\delta \right)}^{d}}{2\times \sigma^{2}}\right)\right), \end{array}$$

where *β*_1_+*β*_2_+*β*_3_=1,  *β* ∈ *R*, and *δ*, *d* > 0.

In this paper, we utilize different values of *β* to mix the three standard kernels (different regions of the input space). In this case, *β* is a vector, i.e., *β*=[*β*_1_, *β*_2_, *β*_3_]. Through this approach, the relative contribution of each kernel to the hybrid kernel, i.e., *G*_lgpk_(*x*_*i*_, *x*_*i*′_), can be easily varied over the input space.

The LGP kernel function takes better global feature extraction ability from the linear kernel, good prediction ability from the polynomial kernel, and better learning ability from the Gaussian kernel. Mercer's theorem provides the necessary and sufficient qualifiers of a valid kernel function. It states that a kernel function is a permissible kernel if the corresponding kernel matrix is symmetric and positive semidefinite (PSD) [5, 38].

A kernel matrix can be validated that it is PSD by determining its spectrum of eigenvalues. It is important to note that a symmetric is positive definite if and only if all its eigenvalues are nonnegative. Considering this, for the proposed kernel to be permissible, it must satisfy Mercer's theorem. This validity can be proved by using the Taylor expansion for the exponential function of equation (13):

(14) $$\begin{array}{c} G_{\text{LGP}}\left(x_{i},x_{i^{\prime }}\right)=\beta_{1}\cdot \left(x_{i}\cdot x_{i^{\prime }}\right)+\beta_{2}\left(-\overset{\infty }{\underset{i=0}{\sum }}\beta_{3}^{i}\cdot \frac{{\left(\eta \times \left(x_{i}\cdot x_{i^{\prime }}\right)+\delta \right)}^{d.i}}{2\times \sigma^{2i}\cdot i!}\right), \end{array}$$

(15) $$\begin{array}{c} G_{\text{LGP}}\left(x_{i},x_{i^{\prime }}\right)=\beta_{1}\left(x_{i}\cdot x_{i^{\prime }}\right)+\beta_{2}\left(-1+\overset{\infty }{\underset{i=1}{\sum }}\frac{-\beta_{3}^{i}}{2\times \sigma^{2i}i!}\left({\left(\eta \left(x_{i}\cdot x_{i^{\prime }}\right)+\delta \right)}^{d.i}\right)\right), \end{array}$$

(16) $$\begin{array}{c} G_{\text{LGP}}\left(x_{i},x_{i^{\prime }}\right)=\beta_{1}\left(x_{i}\cdot x_{i^{\prime }}\right)-\beta_{2}+\beta_{2}\overset{\infty }{\underset{i=1}{\sum }}\frac{-\beta_{3}^{i}}{2\times \sigma^{2i}i!}\left({\left(\eta \left(x_{i}\cdot x_{i^{\prime }}\right)+\delta \right)}^{d.i}\right), \end{array}$$

(17) $$\begin{array}{c} G_{\text{LGP}}\left(x_{i},x_{i^{\prime }}\right)=\beta_{1}\left(x_{i}\cdot x_{i^{\prime }}\right)-\beta_{2}+\beta_{2}\overset{\infty }{\underset{i=1}{\sum }}\frac{-\beta_{3}^{i}}{2\times \sigma^{2i}i!}\cdot K_{\text{Poly}\left(i\right)}, \end{array}$$

(18) $$\begin{array}{c} G_{\text{LGP}}\left(x_{i},x_{i^{\prime }}\right)=\beta_{1}K_{\text{Linear}}-\beta_{2}+\beta_{2}\overset{\infty }{\underset{i=1}{\sum }}\frac{-\beta_{3}^{i}}{2\times \sigma^{2i}i!}\cdot K_{\text{Poly}\left(i\right)}, \end{array}$$

(19) $$\begin{array}{c} G_{\text{LGP}}\left(x_{i},x_{i^{\prime }}\right)=\beta_{1}K_{\text{Linear}}-\beta_{2}+\beta_{2}\overset{\infty }{\underset{i=1}{\sum }}\frac{-\gamma^{i}\times \beta_{3}^{i}}{i!}\cdot K_{\text{Poly}\left(i\right)}, \end{array}$$

where *K*_Poly(*i*)_=(*η*(*x*_*i*_ · *x*_*i*′_)+*δ*)^*d*^ and *K*_Linear_=(*x*_*i*_ · *x*_*i*′_) and *γ*^*i*^=1/(2*∗σ*^2*i*^).

From equation (19), it is evident that *G*_LGP_(*x*_*i*_, *x*_*i*′_) is a mixed kernel comprising of a weighted linear kernel, a constant *β*_2_, and a weighted summation of polynomial kernels. Using propositions (20)–(22) of Theorem 1 and propositions (21) and (22) of Corollary 1 [38], Mercer's conditions are proved to be true for the proposed kernel, and hence, it is a valid kernel.

**Theorem 1 .**

Functions of Mercer's kernels *K*1 and *K*2 are also Mercer's kernels:

(20) $$\begin{array}{c} G\left(x_{i},x_{i^{\prime }}\right)=K1\left(x_{i},x_{i^{\prime }}\right)+K2\left(x_{i},x_{i^{\prime }}\right), \end{array}$$

(21) $$\begin{array}{c} G\left(x_{i},x_{i^{\prime }}\right)=c\cdot K1\left(x_{i},x_{i^{\prime }}\right),\;\text{for all }c\in R^{+}, \end{array}$$

(22) $$\begin{array}{c} G\left(x_{i},x_{i^{\prime }}\right)=K1\left(x_{i},x_{i^{\prime }}\right)+c,\text{ for all }c\in R^{+}. \end{array}$$

**Corollary 1 .**

Functions of a Mercer kernel *K*1 are also Mercer's kernels:

(23) $$\begin{array}{c} G\left(x_{i},x_{i^{\prime }}\right)={\left(K1\left(x_{i},x_{i^{\prime }}\right)+c\right)}^{d},\;\text{ for all }c\in R^{+}\text{ and }d\in N, \end{array}$$

(24) $$\begin{array}{c} G\left(x_{i},x_{i^{\prime }}\right)=\exp\left(\frac{K1\left(x_{i},x_{i^{\prime }}\right)}{\sigma^{2}}\right),\;\text{ for all }\sigma \in R^{+}. \end{array}$$

Since the proposed hybrid LGP kernel combines three valid Mercer's kernels, i.e., linear, Gaussian, and polynomial kernels, it also a valid Mercer's kernel that can be used for training and classification of the multiclass support vector machine (MCSVM).

By using the proposed LGP-MCSVM, the nonlinear transformation of the microarray gene sample points to get the corresponding kernel matrix so as to obtain the classification results during the training phase of the MCSVM classifier.

### 2.5. Particle Swarm Optimization (PSO)

Currently, there is no widely accepted method for optimizing these parameters. The “grid-search (GS)” with exponentially growing sequences of combination {*C*, *η*} for the commonly utilized Gaussian kernel is often applied in microarray analysis [1, 18]. Though it is easy to implement, it has low computing efficiency. In addition, the optimal result of the GS can only be generated from the preset grid combinations while unknown possible optimal parameters cannot be explored and discovered.

In this paper, particle swarm optimization (PSO) optimization technique is adopted to optimally search for the best parameter combinations for the considered models [18, 33]. The PSO technique is derived from the migration patterns of birds during foraging, which has a faster convergence, efficient parallel computing, and a strong universality that is able to efficiently avoid local optimum [20]. In addition, the iteration velocity for its particles is largely influenced by the sum of current velocity, previous particle value, the current global optimal value, and random interferences, which greatly helps avoid the local optimal and improves the search coverage and effectiveness. In order to effectively evaluate the performance of the considered models, different values were considered for all kernel parameters within the following ranges presented in Table 1.

The parameters that need to be determined in the PSO algorithm include the dimension of the search space *D*, the swarm size *n*, cognitive learning factor *c*_1_, social learning factor *c*_2_, the inertia weight *w*, and the maximum number of iterations. The search space dimension *D* for each considered model is equal to the number of parameters required to be set for that model, i.e., PSO + L-MCSVM (*D*=1), PSO + P-MCSVM (*D*=4), PSO + G-MCSVM (*D*=2), and PSO + LGP-MCSVM (*D*=8). Since each model has a different dimensional search space and there is no exact rule in the literature for selecting the swarm size, as a rule of thumb with heuristic optimization algorithms, the swarm size for each model was set to 10 × *D* [39]. According to [40], both the cognitive learning factor and social learning factor were set to 2, i.e., *c*_1_=*c*_2_=2, and the inertia weight *w* was set to 1 as suggested in [41]. To prevent the searches from terminating prematurely and unnecessary additional computational complexity, the maximum number of iterations for all models was set to 50.  Table 2 presents these initial PSO parameters of each model. More information on the PSO algorithm is presented in [18–20, 33, 39–43].

### 2.6. PCA-PSO-LGP-MCSVM Model

The main process of the proposed algorithm is outlined as follows:

1. Transforming the cancer microarray data into the right format for the SVM package.
2. Loading a cancer microarray dataset.
3. Randomly dividing the loaded microarray data into two sets: training set and testing set.
4. Initialize the PSO parameters such as the population size, the maximum number of iterations, and the considered multiclass SVM parameters.
5. Adopt PSO to search for the optimal solution of particles in the global space by using 5-fold cross-validation that incorporates per-fold PCA feature extraction. This process is presented below.
6. To achieve 5-fold cross-validation incorporating PCA, the following steps were followed:
  - For *j* = 1 to 5 repeat steps (ii) to (vi)
  - Carry out PCA on data present in the remaining 4 folds to generate a loadings matrix
  - Transform this data (data in the remaining 4 folds, i.e., calibration set) into a set of principal component (PC) scores using the first *P* components (that account for at least 95% cumulative variance) of the loadings matrix generated in step (ii)
  - Build a considered SVM classification model using a set of parameter values using the generated PC score data in step (iii)
  - Transform the held-out test fold data (i.e., data in fold *j*) into a set of principal component (PC) scores using the *P* component loading matrix retained in step (iii)
  - Compute the classification accuracy of the built SVM classification model in step (iv) using the transformed test fold *j* data in step (v)
  - For the considered parameters set, store their optimal parameter values set (i.e., a set of parameters that yields the highest classification accuracy)
7. Report optimal parameters for the considered model.
8. Carry out PCA on the whole training set data (i.e., the training set obtained in step 3) to generate a loading matrix.
9. Transform this whole training set data into a set of PC scores using the first *P* components (that account for at least 95% cumulative variance).
10. Build an optimal model for the considered SVM classification model using the optimal parameter values set obtained in step (vii) using the generated PC scores data in step 9.
11. Transform the whole testing set data (i.e., the testing set obtained in step 3) into a set of principal component (PC) scores using the *P* component loading matrix retained in step 9.
12. Compute the classification accuracy of the built optimal SVM classification model in step 8 using the transformed whole testing set data in step 9.
13. Report this test classification accuracy.

**Figure 1:**
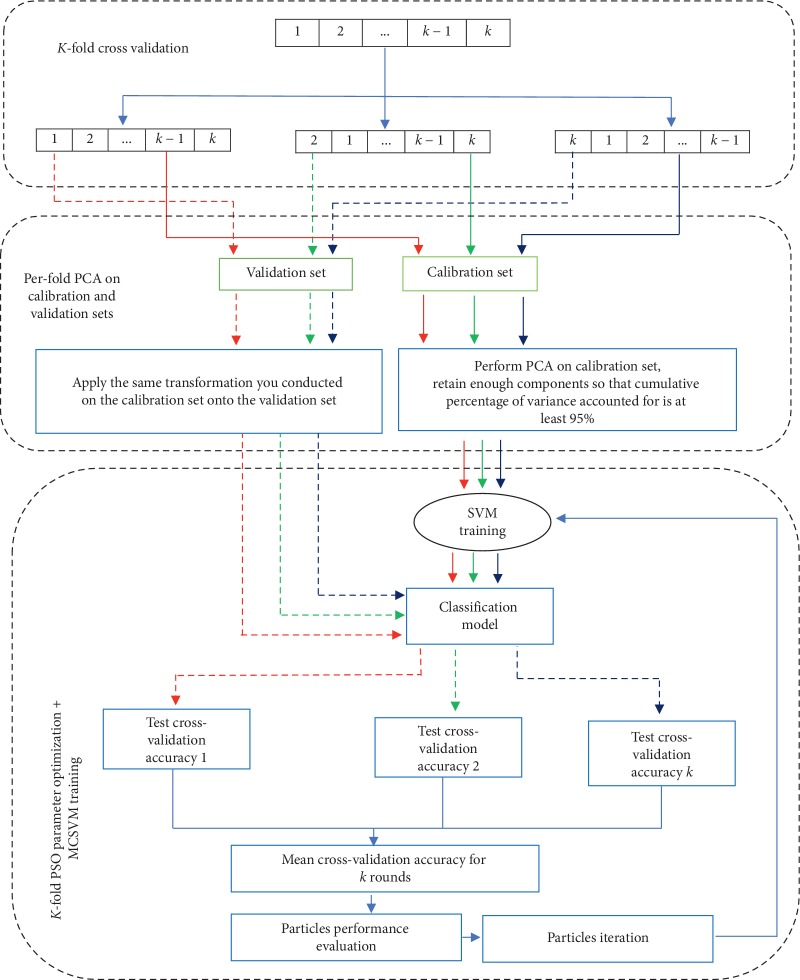
Scheme of the proposed PSO-PCA-LGP-MCSVM algorithm.

The schematic diagram in Figure 1 shows all the process of the PSO-PCA-LGP-MCSVM algorithm.

It is important to mention that the whole analysis process is conducted using the LIBSVM framework in MATLAB [44, 47] on Intel(R) Core (TM) i3-3240M CPU @ 3.4 GHz with 12 GB of RAM machine.

## 3. Performance Evaluation

### 3.1. Considered Microarray Datasets

To assess the performance of the proposed PSO-PCA-LGP-SVM algorithm, several experiments were conducted on four publicly available datasets. Summary of all the datasets utilized in this research can be found in Table 3, and following is a brief description of each dataset:

  Colon dataset [8]: this dataset contains gene expression levels obtained from DNA-based microarrays. It has 62 samples: 22 normal and 40 cancerous tissue samples, each described by 2000 features.
  Leukemia (AML-ALL) dataset [6]: this dataset contains gene expression levels from 72 leukemia patients: 47 with Acute Lymphoblastic Leukemia (ALL) and 25 with Acute Myeloid Leukemia (AML). Each patient data is described by expression levels of 7129 probes obtained from 6817 human genes.
  St. Jude Leukemia dataset [7]: this dataset was obtained from St. Jude Children's Research Hospital. It is divided into 6 diagnostic groups: BCR-ABL (9 patients), E2A-PBX1 (18 patients), Hyperdiploid > 50 (42 patients), Mixed Lineage Leukemia(MLL) (14 patients), T-cell Acute Lymphoblastic Leukemia (T-ALL) (28 patients), and TEL-Leukemia (TEL-AML1) (52 patients) and other 52 patients that could not fit into any of the outlined diagnostic groups. This dataset contains 12558 genes.
  Lung Cancer dataset [13]: this dataset contains 3312 gene data obtained from 17 people with normal lungs and 186 lung cancer patients that is classified into 5 classes: Adenocarcinomas (139 patients), Squamous Cell Lung Carcinomas (21 patients), Pulmonary Carcinoids (20 patients), Small Cell Lung Carcinomas (6 patients), and Normal Lung (17 people).

Due to the small number of instances in the considered datasets, all the datasets were initially split into two disjoint sets: the training set and the test set. Utilizing 5-fold cross-validation, the training set was randomly divided further into 5 subsets (approximately) equal in size. Each time 4 subsets were selected as the calibration set and the remaining subset was used as the validation set. This process was repeated 5 times. Finally, the average of classification accuracy on the validation set was used as one of the evaluation metrics. It is important to point out that by using 5-fold cross-validation to dynamically divide the microarray training samples, the considered models turn out to be more stable and objective.

The percentage proportion for the calibration, validation, and test sets for all the considered microarray datasets is presented in Table 4.

### 3.2. Performance Measures for Imbalanced Microarray Datasets

When the samples in a dataset are unevenly distributed among the classes (for instance, in the case of microarray datasets), the task of classification in imbalanced domains must be defined. The majority class, as a result, influences the data mining algorithms skewing their performances towards it [15].

Most algorithms simply compute the accuracy on the basis of the percentage of correct samples.

However, in the case of microarrays, these results are highly deceiving since the minority classes hold minimal effects on the overall classification accuracy. Thus, a consideration of a complete confusion matrix (Table 5) must be made to obtain the classification of both positive and negative classes independently [15].

The description in Table 5 gives four baseline statistical components, where TP and FN denote the number of positive samples, which are accurately and falsely predicted, respectively, and TN and FP depict the number of negative samples that are predicted accurately and wrongly, respectively.

Two most frequently used metrics for class imbalance problem, namely, *F*-measure and *G*-mean, can be regarded as functions of these four statistical components and are calculated as follows:

(25) $$\begin{array}{c} F-\text{measure}=\frac{2*\text{Recall}*\text{Precision}}{\left(\text{Recall}+\text{Precision}\right)}, \end{array}$$

(26) $$\begin{array}{c} G-\text{mean}=\sqrt{\left(\text{TPR}\times \text{TNR}\right)}, \end{array}$$

where precision, recall, TPR, and TNR are further defined as follows:

(27) $$\begin{array}{c} \text{Precision}=\frac{\text{TP}}{\left(\text{TP}+\text{FP}\right)},\\ \text{Recall }\left(\text{TPR}\right)=\frac{\text{TP}}{\left(\text{TP}+\text{FN}\right)},\\ \text{TNR}=\frac{\text{TN}}{\left(\text{TN}+\text{FP}\right)}. \end{array}$$

The overall classification accuracy (Acc) can be calculated using the following equation:

(28) $$\begin{array}{c} \text{Acc}=\frac{\text{TP}+\text{TN}}{\left(\text{TP}+\text{TN}+\text{FP}+\text{FN}\right)}. \end{array}$$

However, all these evaluation metrics are appropriate for estimating binary-class imbalance tasks. To extend them for multiclass, the following transformations should be considered [15].*G*-mean computes the geometric mean of all the classes' accuracies and is defined by

(29) $$\begin{array}{c} G-\text{mean }={\left(\overset{C}{\underset{i=1}{\prod }}{\text{Acc}}_{i}\right)}^{1/C}, \end{array}$$

where Acc_*i*_ denotes the accuracy of the *i*^th^ class.  *F* − measure can be transformed as *F*-score and is computed using the following equation:

(30) $$\begin{array}{c} F-\text{Score }=\frac{\sum_{i=1}^{C}F{-\text{measure}}_{i}}{C}, \end{array}$$

where *F*−measure_*i*_ is calculated further using the following equation:

(31) $$\begin{array}{c} F{-\text{measure}}_{i}\;=\frac{2\times {\text{Precision}}_{i}\times {\text{Recall}}_{i}}{{\text{Precision}}_{i}+{\text{Recall}}_{i}}. \end{array}$$

Acc can be transformed as depicted by the following equation:

(32) $$\begin{array}{c} \text{Acc }=\overset{C}{\underset{i=1}{\sum }}\left({\text{Acc}}_{i}\times P_{i}\right), \end{array}$$

where *P*_*i*_ is the percentage of samples in the *i*^th^ class. To impartially and comprehensively assess the classification performance of the proposed model in comparison with PSO-PCA-L-MCSVM, PSO-PCA-G-MCSVM, and PSO-PCA-P-MCSVM models that utilize the standard linear, Gaussian, and polynomial kernels, respectively, the three extended measures, namely, *F*-score, *G*-mean, and Acc which are described in (29), (30), and (32), respectively.

## 4. Results and Discussions

The experimental results for the 4 classification models on the 4 microarray datasets are reported in Tables 6–8, where the best result in each dataset is highlighted in bold and the worst is italicized.

From Tables 6–8, the following observations can be made

- Lung and St. Jude datasets are slightly sensitive to the class imbalance while Colon and AML-ALL are not, as shown by the difference between Accuracy and *G*-mean values. An Accuracy slightly lower than the G-mean values implies that the MCSVM is affected by the imbalanced class distribution. This is largely attributed by a large number of true negatives (TNs) recorded achieved by all the models when analyzing both the Lung and St. Jude datasets.
- The hybrid kernel boosted the classification performance of the multiclass on three datasets, i.e., Colon, Lung, and St. Jude. These promotions are better portrayed by the *F*-score and *G*-mean metrics, which are used to evaluate a balance level of classification results. However, a tie is reported for the AML-ALL dataset. This implies that though the complementary characteristics of the three standard kernels, i.e., linear, Gaussian, and polynomial, in the proposed hybrid linear-Gaussian-polynomial (LGP) kernel may improve the multiclass support vector machine classifier's classification ability on most microarray datasets, a single suitable kernel is sufficient.
- Of all the considered models, the PSO-PCA-P-MCSVM reported the least performance in all the considered metrics for all the four datasets. However, it is important to note that a promising kernel can be obtained if we embed into the exponential kernel.

In summary, compared with single kernel-based models (i.e., PSO-PCA-L-MCSVM, PSO-PCA-G-MCSVM, and PSO-PCA-P-MCSVM), the proposed PSO-PCA-LGP-MCSVM model that is based on a hybrid linear-Gaussian-polynomial (LGP) kernel with a better global feature extraction ability, good prediction ability, and better learning ability, has an attractive classification ability in cancer diagnosis using both imbalanced dual and multiclass microarray datasets. Moreover, due to the excellent global searching ability of the particle swarm optimization, it can effectively optimize the hybrid kernel-based MCSVM when solving a wider range of classification problems.

## 5. Conclusion

Techniques to choose or construct suitable kernel functions and optimally tune its parameters for MCSVM have received a considerable and critical attention in imbalanced microarray-based cancer diagnosis. A novel classification model, PSO-PCA-LGP-MCSVM, that is based on MCSVM with a hybrid kernel, i.e., linear-Gaussian-polynomial (LGP), is proposed in this paper. The LGP kernel combines the advantages of three standard kernels, i.e., linear, Gaussian, and polynomial kernels in a novel manner where the linear kernel is linearly combined with a polynomial kernel that is embedded into a Gaussian kernel. Using PSO to optimally tune the LGP kernel-based MCSVM resulted in better generalization, learning, and predicting ability as evidenced by the promising results in terms of three extended measures *F*-score, *G*-mean, and Accuracy irrespective of imbalanced binary or multiclass microarray datasets. The performance of the proposed model was compared with those of 3 models, i.e., PSO-PCA-L-MCSVM, PSO-PCA-G-MCSVM, and PSO-PCA-P-MCSVM that are based on single linear, Gaussian, and polynomial kernels, respectively, and the experimental results show that the proposed model is superior to the three single-kernel-based models. This reflects the good practical value of the proposed model in the field of microarray-based cancer diagnosis, which can also be extended to more applications of medical diagnostic classification to explore its potential.

## Figures and Tables

**Table 1:** Parameters and their respective ranges.

Parameter	Range
*β*=[*β*_1_, *β*_2_, *β*_3_]	0 < *β*_1_, *β*_2_, *β*_3_ < 1 and *β*_1_ + *β*_2_ + *β*_3_ = 1
log_2_ *C*	−5 ≤ log_2_ *C* ≤ 15
*δ*	0 ≤ *δ* ≤ 5
*d*	2 ≤ *d* ≤ 5
log_2_ *γ*, log_2_ *η*	−15≤ log_2_ *γ*, log_2_ *η* ≤ 3

**Table 2:** Initial PSO parameters setting.

Parameter	Range
Maximum number of iterations	50
Inertial weight, *w*	1
Number of particles/swarm size	(1) PSO + L-MCSVM = 10
	(2) PSO + G-MCSVM = 20
	(3) PSO + *P*-MCSVM = 40
	(4) PSO + LGP-MCSVM = 80
Cognition learning factor, *c*_1_	2.0
Social learning factor, *c*_2_	2.0

**Table 3:** The cancer microarray datasets utilized in this paper.

Category	Dataset	Sample size	Number of genes	Number of classes
Two-class	AML-ALL	72	7129	2
	Colon	62	2000	2
Multiclass	St. Jude	215	12558	7
	Lung	203	3312	5

**Table 4:** Percentage proportion for the calibration, validation, and test sets.

Dataset	% proportion for calibration set	% proportion for validation set	% proportion for test set
AML-ALL	61.1	15.3	23.6
Colon	58.1	14.5	27.4
St. Jude	57.7	14.4	27.9
Lung	57.1	14.3	28.6

**Table 5:** Confusion matrix for a two-class problem.

	Positive prediction	Negative prediction
Positive class	True positive (TP)	False negative (FN)
Negative class	False positive (FP)	True negative (TN)

**Table 6:** Accuracy of all considered models on the four microarray datasets.

Models	Colon	Lung	AML-ALL	St. Jude
PSO + L-MCSVM	*0.7647*	0.9596	**0.9412**	0.9422
PSO + P-MCSVM	0.8235	*0.9592*	*0.8235*	*0.9395*
PSO + G-MCSVM	0.8235	0.9608	0.9412	0.9572
PSO + LGP-MCSVM	**0.8824**	**0.9729**	**0.9412**	**0.9603**

Values in bold represent the best result and values in italic denote the worst in each column, respectively.

**Table 7:** *F*-score of all considered models on the four microarray datasets.

Models	Colon	Lung	AML-ALL	St. Jude
PSO + L-MCSVM	*0.7572*	0.9246	0.9328	0.7870
PSO + P-MCSVM	0.8211	*0.7524*	*0.7733*	*0.6831*
PSO + G-MCSVM	0.8211	0.9306	**0.9377**	0.8477
PSO + LGP-MCSVM	**0.8712**	**0.9586**	**0.9377**	**0.8989**

Values in bold represent the best result and values in italic denote the worst in each column, respectively.

**Table 8:** *G*-mean of all considered models on the four microarray datasets.

Models	Colon	Lung	AML-ALL	St. Jude
PSO + L-MCSVM	*0.7676*	0.9791	**0.9412**	0.9557
PSO + P-MCSVM	0.8235	*0.7524*	*0.8235*	*0.9512*
PSO + G-MCSVM	0.8235	0.9792	**0.9412**	0.9661
PSO + LGP-MCSVM	**0.8824**	**0.9861**	**0.9412**	**0.9709**

Values in bold represent the best result and values in italic denote the worst in each column, respectively.

## Data Availability

The data used to support the findings of this study are available from the corresponding author upon request.

## Supplementary Materials

Supplementary Materials The results presented in Tables 6–8 are based on confusion matrices attached as supplementary materials whereby Figure 1, Figure 2, Figure 3, and Figure 4 represent the confusion matrices obtained when the trained PSO-PCA-L-MCSVM, PSO-PCA-G-MCSVM, PSO-PCA-P-MCSVM, and PSO-PCA-LGP-MCSVM models were evaluated using the Colon, Lung, AML-ALL, and St. Jude test set samples, respectively.
