# Supplementary material for: Particle Swarm Optimized Hybrid Kernel-Based Multiclass Support Vector Machine for Microarray Cancer Data Analysis
Source: Biomed Res Int. 2019 Dec 14;2019:4085725. doi: 10.1155/2019/4085725 (PMC6973196; doi:10.1155/2019/4085725)
Supplement: Supplementary Materials — The results presented in Tables 6–8 are based on confusion matrices attached as supplementary materials whereby Figure 1, Figure 2, Figure 3, and Figure 4 represent the confusion matrices obtained when the trained PSO-PCA-L-MCSVM, PSO-PCA-G-MCSVM, PSO-PCA-P-MCSVM, and PSO-PCA-LGP-MCSVM models were evaluated using the Colon, Lung, AML-ALL, and St. Jude test set samples, respectively. [file 4085725.f1.pdf]

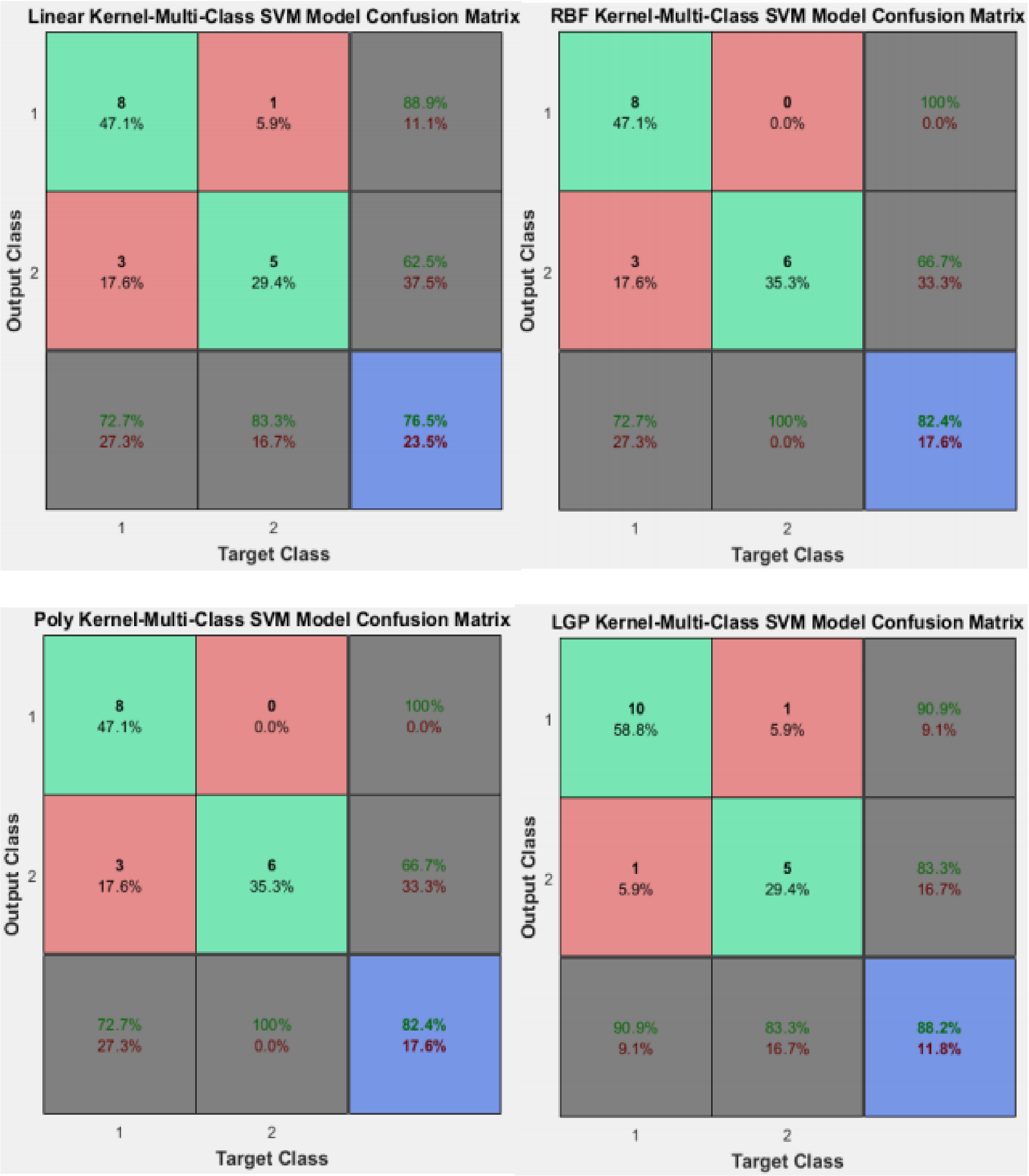

Figure 1: PSO-PCA-L-MCSVM, PSO-PCA-RBF-MCSVM, PSO-PCA-P-MCSVM and PSO-PCA-LGP-MCSVM confusion matrices for the Colon Cancer test samples

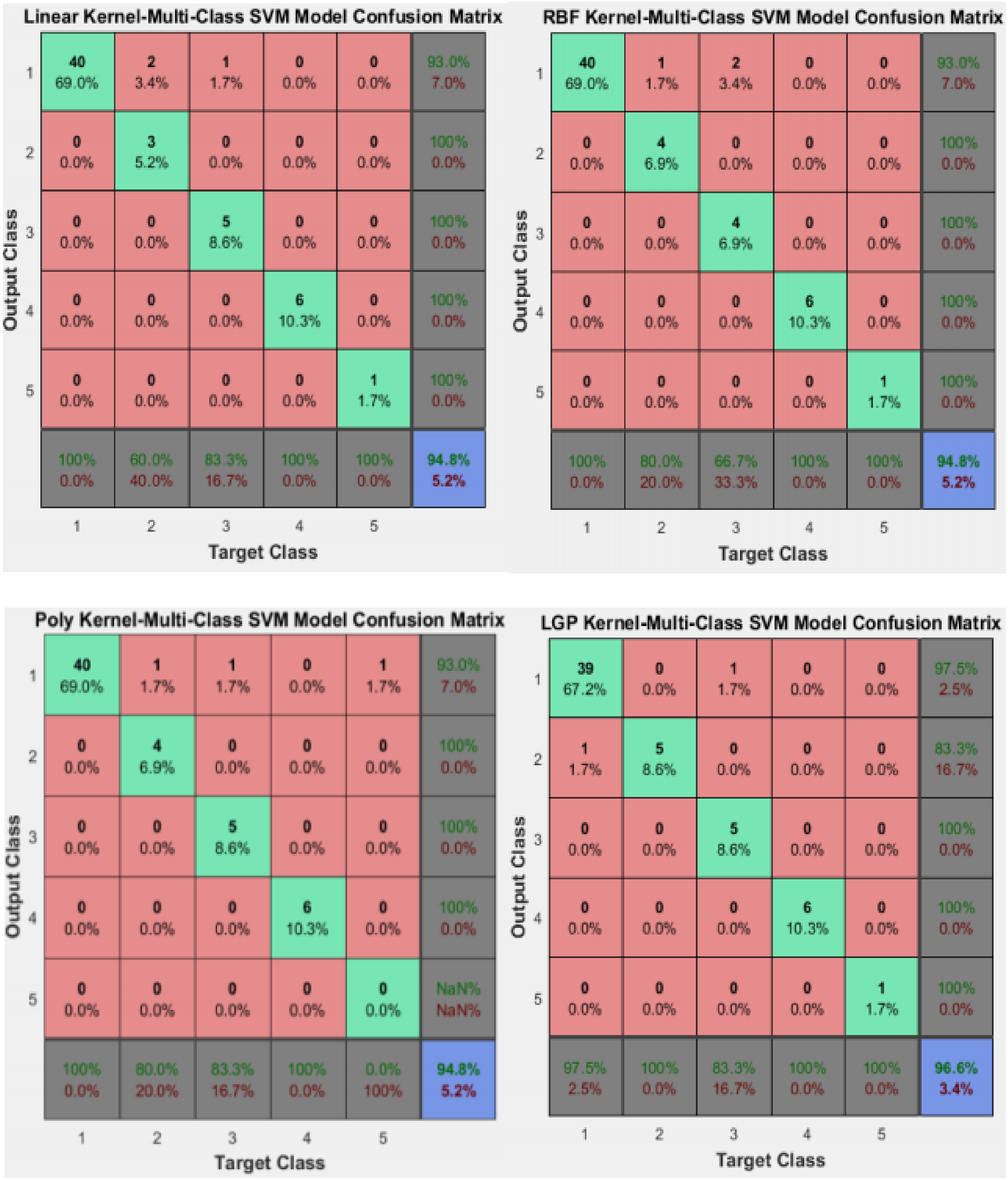

Figure 2: PSO-PCA-L-MCSVM, PSO-PCA-RBF-MCSVM, PSO-PCA-P-MCSVM and PSO-PCA-LGP-MCSVM confusion matrices for the LUNG Cancer test samples

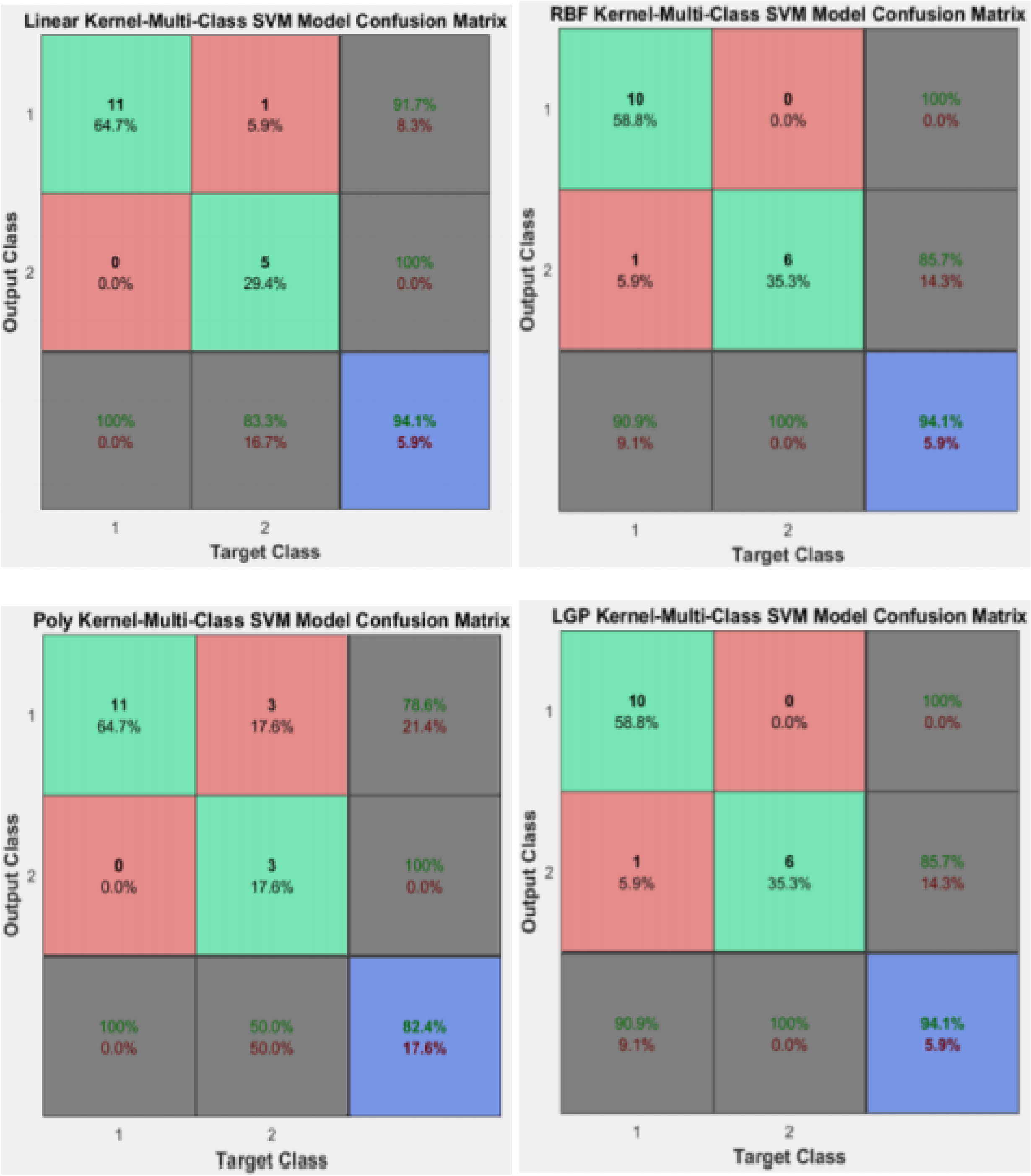

Figure 3: PSO-PCA-L-MCSVM, PSO-PCA-RBF-MCSVM, PSO-PCA-P-MCSVM and PSO-PCA-LGP-MCSVM confusion matrices for the AMLALL test samples

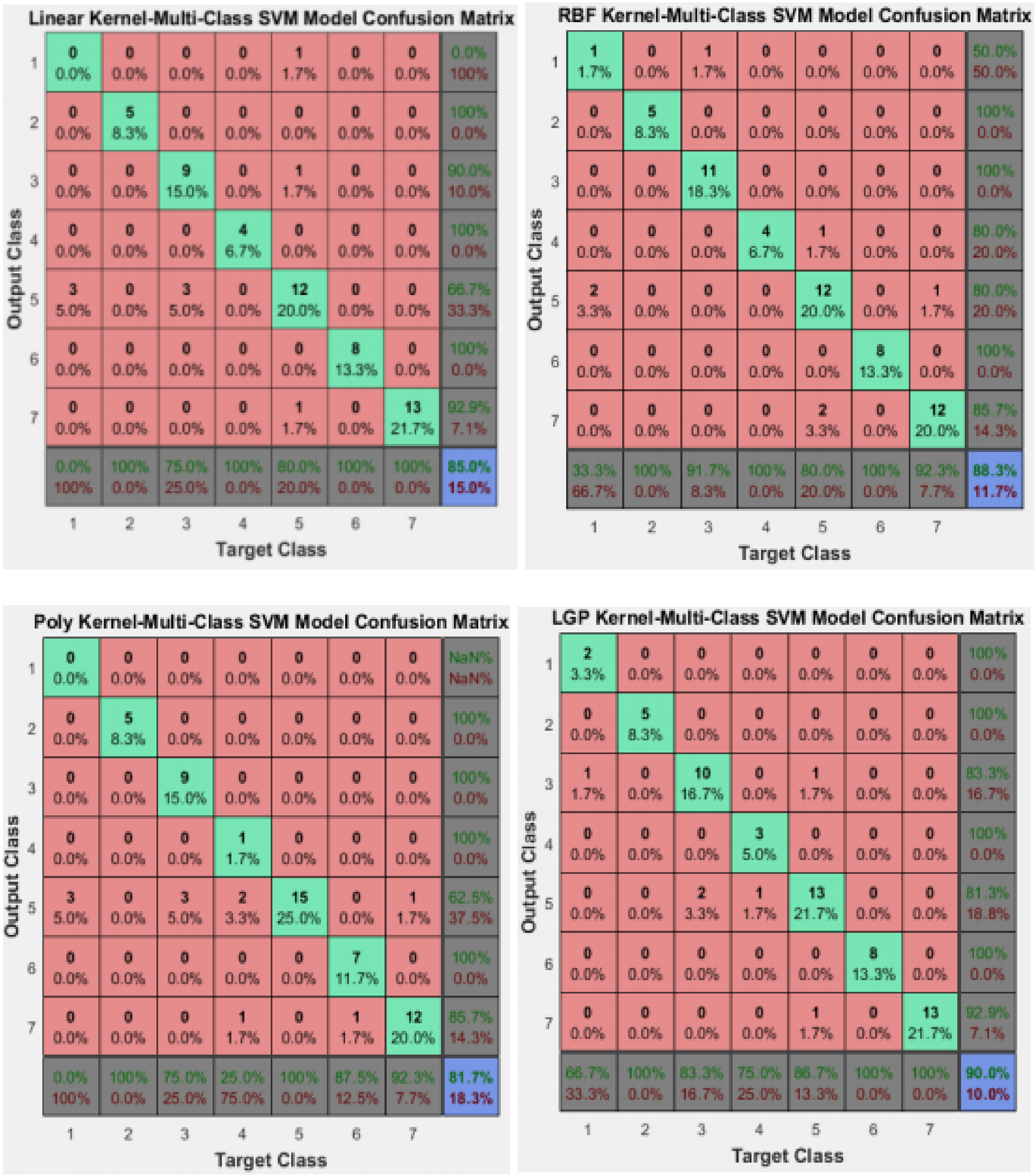

Figure 4: PSO-PCA-L-MCSVM, PSO-PCA-RBF-MCSVM, PSO-PCA-P-MCSVM and PSO-PCA-LGP-MCSVM confusion matrices for the STJUDE test samples
